# Barium Concentration-Dependent Anomalous Electrophoresis of Synthetic DNA Motifs

**DOI:** 10.1021/acsabm.4c00274

**Published:** 2024-04-18

**Authors:** Bharath Raj Madhanagopal, Arlin Rodriguez, Mireylin Cordones, Arun Richard Chandrasekaran

**Affiliations:** † The RNA Institute, University at Albany, State University of New York, Albany, New York 12222, United States

**Keywords:** DNA nanostructures, metal ions, barium, electrophoresis, self-assembly, gel mobility

## Abstract

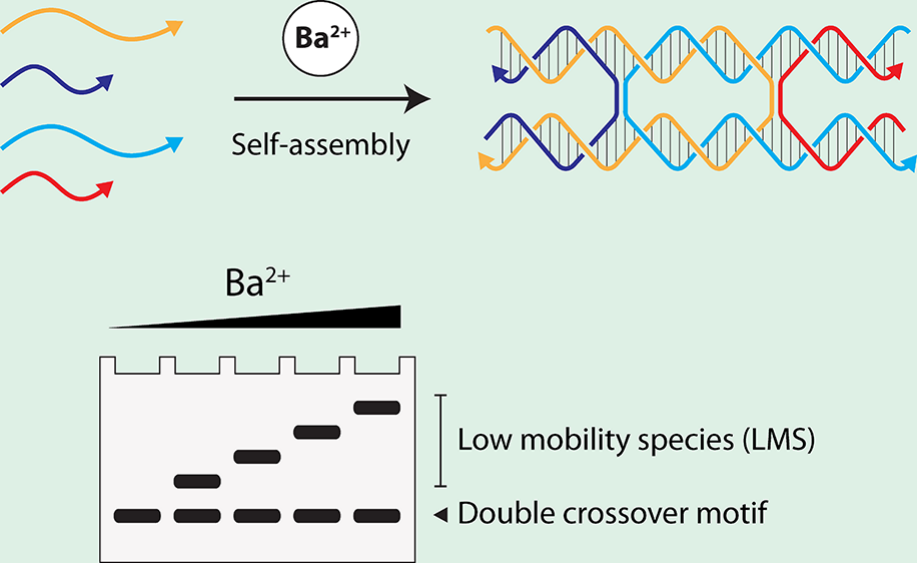

The structural integrity, assembly yield, and biostability of DNA nanostructures are influenced by the metal ions used to construct them. Although high (>10 mM) concentrations of divalent ions are often preferred for assembling DNA nanostructures, the range of ion concentrations and the composition of the assembly products vary for different assembly conditions. Here, we examined the unique ability of Ba^2+^ to retard double crossover DNA motifs by forming a low mobility species, whose mobility on the gel is determined by the concentration ratio of DNA and Ba^2+^. The formation of this electrophoretically retarded species is promoted by divalent ions such as Mg^2+^, Ca^2+^, and Sr^2+^ when combined with Ba^2+^ but not on their own, while monovalent ions such as Na^+^, K^+^, and Li^+^ do not have any effect on this phenomenon. Our results highlight the complex interplay between the metal ions and DNA self-assembly and could inform the design of DNA nanostructures for applications that expose them to multiple ions at high concentrations.

Assembly of nanostructures using DNA has relied on the highly predictable nature of the canonical Watson–Crick–Franklin base-pairing and the well-known geometry of B-DNA.^1,2^ Because of their highly anionic nature, assembly of DNA strands into multihelical-domain nanostructures generally requires the use of cations. The structural and dynamic properties of DNA double helices within such nanostructures are influenced by the interaction of the DNA strands with these ions.^3^ While Mg^2+^ is widely used for DNA self-assembly, other divalent ions (e.g., Ca^2+^, Ba^2+^, and Sr^2+^) and monovalent ions (e.g., Na^+^, Li^+^, and K^+^) have also been used for this purpose.^4−7^ In contrast to the “universal” use of Mg^2+^, the success of using other cations for DNA self-assembly has varied, depending on the type or complexity of the DNA nanostructure and the ion concentration used. Since the assembly of nanostructures and their structural properties depend on the nature and concentration of the metal ion used it is imperative to understand the effect of different ions and their combinations on nanostructure assembly. In our recent work, we demonstrated the assembly of a double crossover DNA motif using a variety of cations, typically used at 10 mM concentration.^6^ When testing assembly of the double crossover DNA motif at higher ion concentrations, we observed the formation of what appeared to be higher molecular weight species in nondenaturing polyacrylamide gels at Ba^2+^ concentrations of >25 mM, but not for similar concentrations of Mg^2+^ or Ca^2+^. Interestingly, the mobility of the band was dependent on the concentration of Ba^2+^. In this study, we investigated the nature of this species using gel electrophoresis (Figure 1a) and spectroscopic methods to better understand the role of Ba^2+^ in the assembly of nanostructures and the impact of the combinations of metal ions on DNA self-assembly.

**Figure 1:**
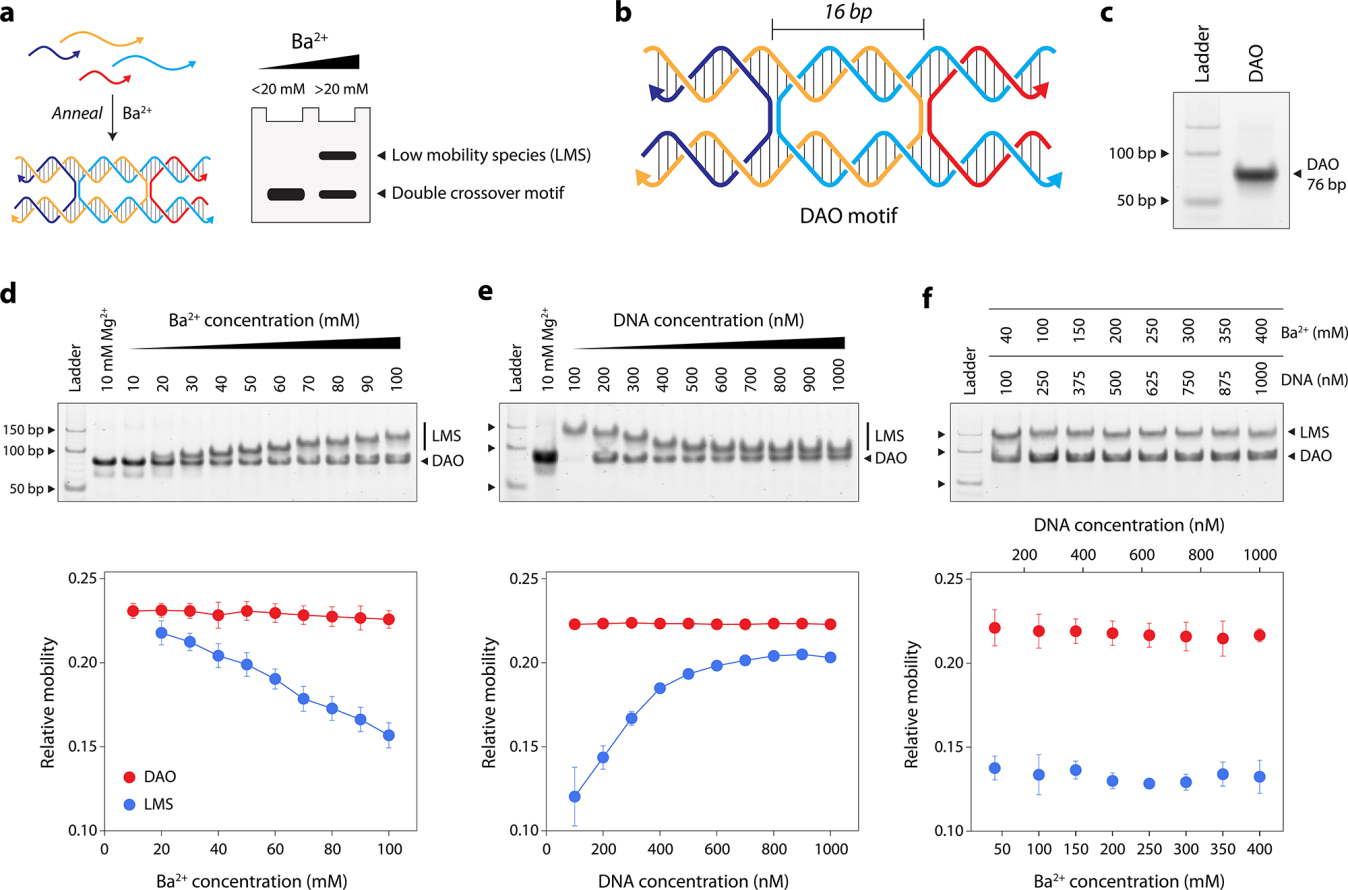
Ba^2+^ induced formation of low mobility species. (a) Schematic of DNA motif assembly in Ba^2+^ and analysis of low mobility species (LMS). (b) Design of the double crossover antiparallel motif with an odd number of half turns between crossovers (DAO). (c) Nondenaturing gel image showing typical assembly of DAO in TAE-Mg^2+^ buffer. (d) Nondenaturing gel showing the formation of LMS in 250 nM DAO assembled in 1× TAE containing different concentrations Ba^2+^. Relative mobility of DAO and LMS at each Ba^2+^ concentration is also shown. (e) Nondenaturing gel and relative mobility analysis of DAO and LMS at fixed Ba^2+^ concentration (100 mM) and different DNA concentrations. (f) Nondenaturing gel and relative mobility analysis of DAO and LMS at fixed [DAO]:[Ba^2+^] ratios from 100 nM DNA:40 mM Ba^2+^ to 1000 nM DNA:400 mM Ba^2+^.

To study the effects of Ba^2+^ on DNA nanostructure assembly, we chose the double crossover DNA motif as a model structure (Figure 1b and Figure S1).^8^ The double crossover DNA motif we use here contains two double helical domains connected by two antiparallel crossovers separated by an odd number of half turns (16 bp, abbreviated DAO). We validated assembly of the DAO motif in the typically used tris-acetate-EDTA (TAE) buffer containing ∼10 mM Mg^2+^ using nondenaturing polyacrylamide gel electrophoresis (PAGE) (Figure 1c). We then assembled the DAO motif at a fixed DNA concentration (250 nM DAO) in TAE buffer containing different concentrations of Ba^2+^ and characterized the samples using nondenaturing PAGE (Figure 1d, full gel image in Figure S2). At 10 mM Ba^2+^, a single band with a mobility expected for a 76 bp structure was observed, matching the band corresponding to the DAO assembled in 10 mM Mg^2+^. At higher concentrations of Ba^2+^, another band with a lower mobility was observed. The mobility of this band decreased linearly with increasing concentrations of Ba^2+^. We then tested whether the concentration of DNA in solution had any effect on the appearance of this low mobility species (LMS). We varied the concentration of the DAO motif from 100 nM to 1000 nM while keeping the concentration of Ba^2+^ constant at 100 mM, annealed the samples and analyzed them using nondenaturing PAGE (Figure 1e, full gel image in Figure S3). We observed the formation of the LMS in all of the combinations. However, with increasing concentrations of DAO, the relative mobility (*R*_f_) of the LMS increased until it had a similar *R*_f_ to that of the DAO band at 1000 nM DAO and 100 mM Ba^2+^. We then chose the highest concentration of DAO (1000 nM) and tested assembly with higher Ba^2+^ concentrations to check whether this trend occurs at higher DNA concentrations. We observed the appearance of LMS with the lowest *R*_f_ in the 1000 nM DAO and 400 mM Ba^2+^ sample (Figure S4). To confirm that the position of the LMS band depends on the concentration ratio of [DAO]:[Ba^2+^] rather than the concentration of DNA or Ba^2+^ alone, we changed the concentration of DAO motif from 100 to 1000 nM and proportionally increased the concentration of Ba^2+^ to maintain the same [DAO]:[Ba^2+^] ratio (from 100 nM DNA:40 mM Ba^2+^ to 1000 nM DNA:400 mM Ba^2+^). The *R*_f_ remained constant in all of the samples, confirming that the mobility of the LMS is determined by the concentration ratio of DNA and the Ba^2+^ in the buffer (Figure 1f, full gel image in Figure S5). This phenomenon was unique to Ba^2+^ and did not occur when other divalent alkali earth metal ions Mg^2+^, Ca^2+^, and Sr^2+^ were used at such high concentrations in the annealing buffer (Figure S6).

We then characterized the electrophoretic mobility of the LMS as a function of polyacrylamide concentration using a Ferguson plot (Figure 2a and Figure S7a).^9,10^ The log *R*_f_ changes linearly with the percentage of acrylamide and the slope of this line represents the retardation coefficient *K*_R_. We observed that the Y-intercept for the LMS decreased with an increasing concentration of Ba^2+^ (Figure S7b). However, the retardation coefficient of the LMS did not change with the concentration of Ba^2+^ in the buffer, and this value was similar to that of the DAO motif. The band corresponding to the DAO structure did not show any variation in either the slope or the Y-intercept across the Ba^2+^ concentrations tested here (Figure S7c). Such trends in biomacromolecules have previously been associated with changes in the overall charge of the molecules,^9^ suggesting that the various low mobility species formed at different concentrations of Ba^2+^ are complexes of DAO with different numbers of bound Ba^2+^ ions. To investigate whether the formation of the LMS is accompanied by a conformational change in the DAO motif, we recorded circular dichroism (CD) spectra of the DAO motif assembled in different concentrations of Ba^2+^ (Figure 2b). The DAO motif assembled with 10 mM Mg^2+^ showed a negative band at 245 nm and a positive band at 275 nm, consistent with earlier reports.^6^ The CD signatures of the DAO assembled with Ba^2+^ did not differ from that assembled with Mg^2+^, indicating that the underlying B-form structure of the DNA motif is largely unaltered by the Ba^2+^ ions both in the DAO and in the LMS. Following this, we investigated the thermal melting profiles of the DAO motif assembled in Mg^2+^ and different concentrations of Ba^2+^ (Figure 2c). DAO motif assembled with 10 mM Mg^2+^ showed a melting temperature (*T*_m_) of 68 °C while the DAO motif assembled with 10 mM Ba^2+^ showed a *T*_m_ of 63.1 °C. An increase in the concentration of the Ba^2+^ to 100 mM (at 1000 nM DNA concentration) increased the *T*_m_ by 2.5 °C, and a further increase in the ion concentration did not change the thermal stability of the structure substantially (Table S2). All the samples showed a single sigmoidal transition indicating that the LMS is not a distinct thermally stable structure.

**Figure 2:**
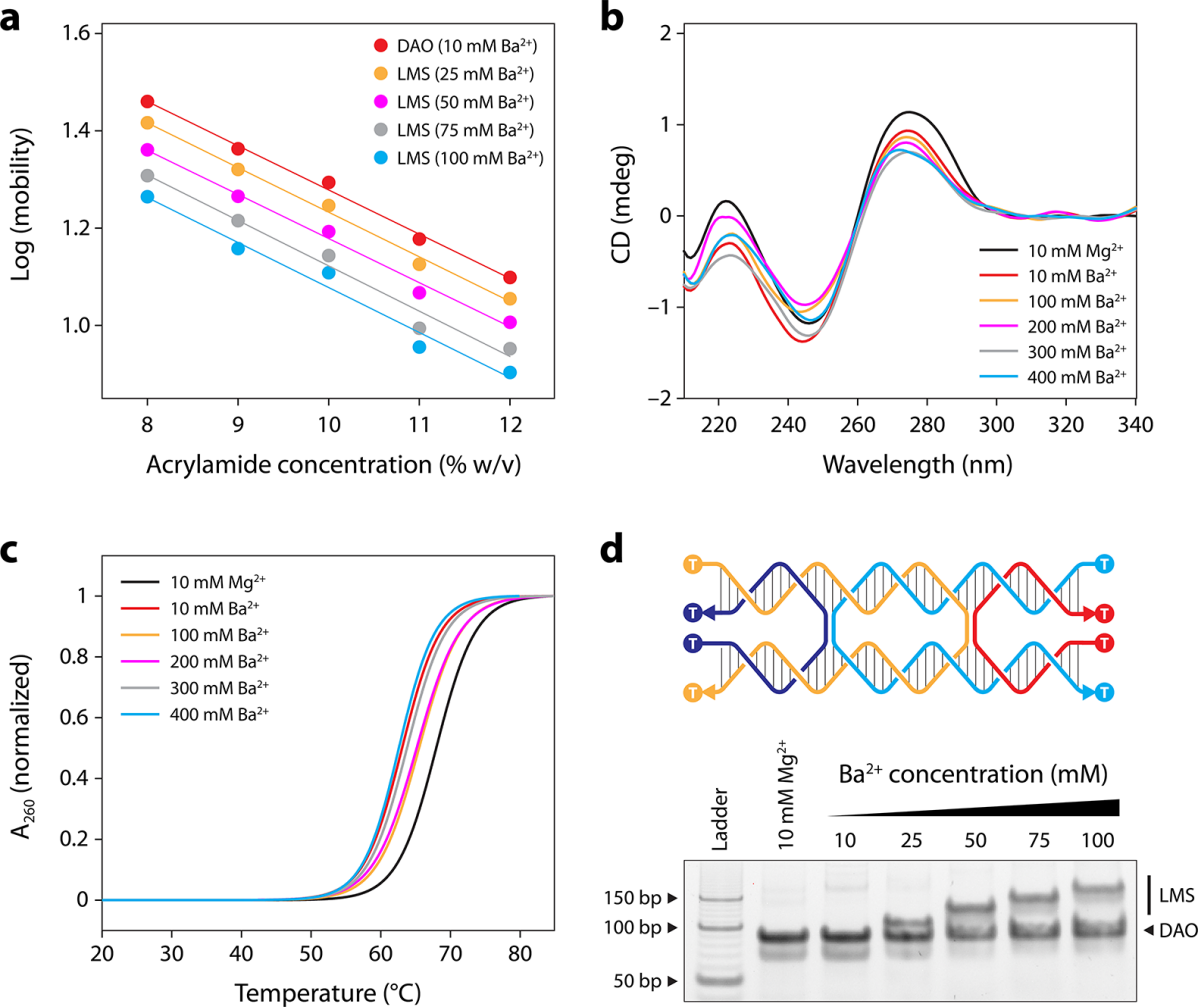
Characterization of the low mobility species. (a) Ferguson plot of DAO and LMS at different Ba^2+^ concentrations. (b) Circular dichroism spectra of DAO assembled in different concentrations of Ba^2+^. (c) UV melting curves of DAO assembled in 10 mM Mg^2+^ and in 10–400 mM Ba^2+^ concentrations. (d) Top: Design of the DAO motif with terminal thymines. Bottom: Nondenaturing gel showing the formation of LMS in DAO with terminal thymines assembled in 1× TAE containing 25–100 mM Ba^2+^.

Construction of DNA motifs has, in some instances, resulted in the formation of higher order assemblies or aggregates. In several cases, addition of terminal thymines on the component DNA strands has reduced such aggregation.^11−14^ To test whether the LMS consists of higher order assemblies of the DAO motif, we designed a DAO motif containing unpaired thymines at the termini of all four strands to disrupt any base stacking interactions that could stabilize lateral interactions between the motifs (Figure S8). We assembled this motif in TAE buffer containing different concentrations of Ba^2+^ and characterized it using nondenaturing PAGE (Figure 2d, full gel image in Figure S9). The gel migration properties of the DAO motif with terminal thymines at different Ba^2+^ concentrations resembled those of the DAO and formation of the LMS was not prevented by the free thymines at the termini, suggesting that the LMS is not an aggregate formed by end-to-end interaction of DAO motifs.

While the formation of LMS is seen at high concentrations of only Ba^2+^ and none of the other commonly used monovalent and divalent ions exhibit this phenomenon (Figure S6), we tested whether the presence of these other ions in combination with Ba^2+^ would promote or reduce the process of LMS formation. We assembled the DAO motif in TAE buffer containing Ba^2+^ and one other monovalent (Li^+^, Na^+^, and K^+^) or divalent metal ion (Mg^2+^, Ca^2+^, and Sr^2+^) and analyzed the samples using nondenaturing PAGE (Figure 3a, full gels in Figures S10–S15). The presence of two different metal ions did not affect the *R*_f_ of the DAO band. To assess the influence of these metal ions on the retardation of the LMS, we calculated the change in *R*_f_ of the LMS band in different ion combinations relative to its position in samples with only Ba^2+^ (Figure 3b). The addition of monovalent ions did not affect the retardation of the LMS, but divalent ions reduced the mobility of the LMS bands further to different extents (Figure 3c). This effect in divalent ions was more prominent at lower concentrations of Ba^2+^. Combination of Sr^2+^ and Ba^2+^ showed a steady Sr^2+^-concentration-dependent retardation of the LMS band when the Ba^2+^ concentration was 25–75 mM (Figure 3b). Mg^2+^ showed a similar effect, but produced more diffused LMS bands when the concentration of Ba^2+^ was 25 mM. In samples containing Ca^2+^ and Ba^2+^, the bands appeared to be more diffuse suggesting the loss of structural integrity or aggregation when these two ions are present together. Collectively, these results show that although other divalent ions (Mg^2+^, Sr^2+^, and Ca^2+^ tested here) were unable to produce the LMS band on their own at 100 mM (Figure S6), they influenced the retardation of the DAO and generate LMS in combination with Ba^2+^.

**Figure 3:**
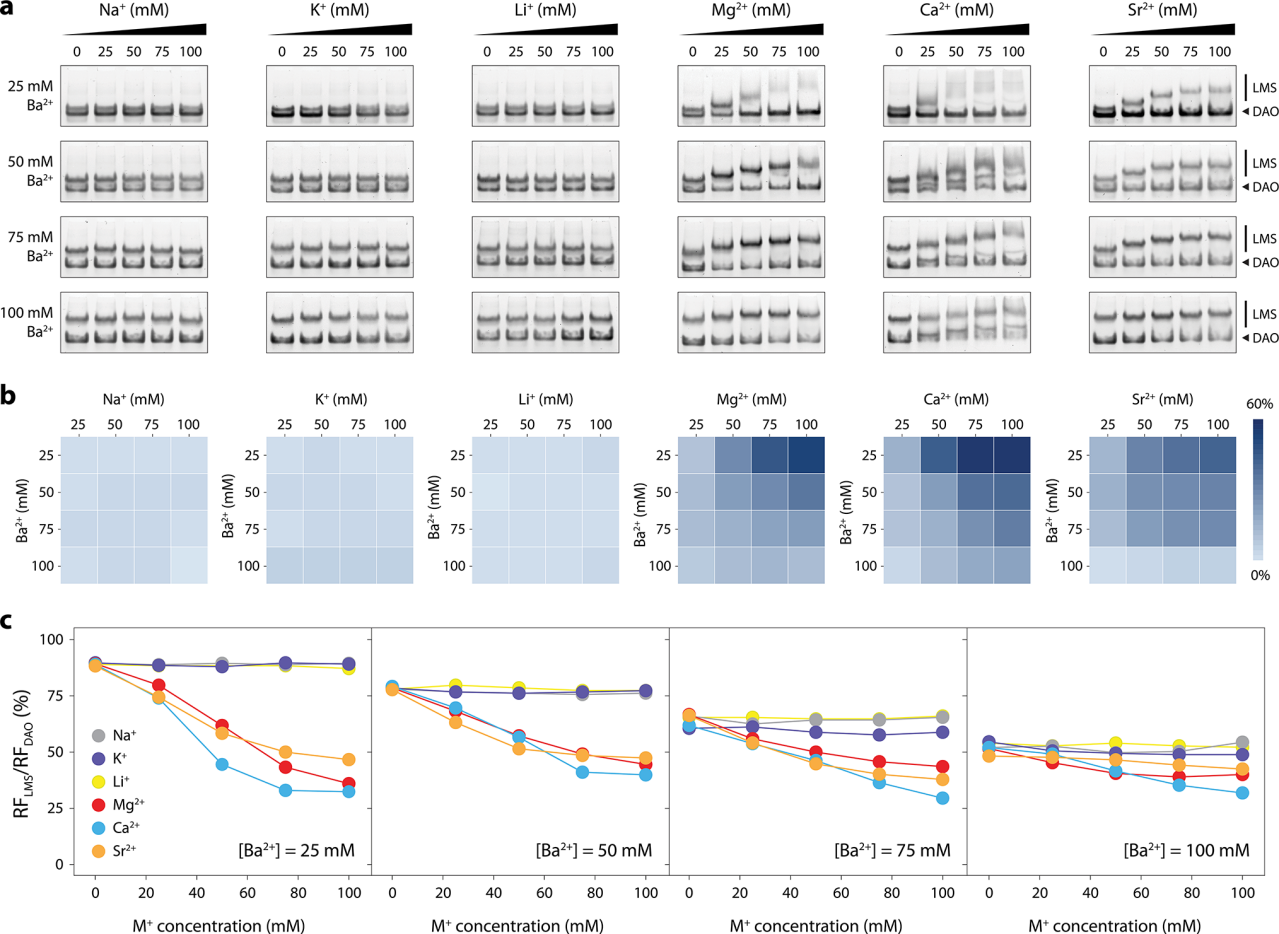
Effect of other metal ions on the formation of low mobility species. (a) Nondenaturing gels showing DAO assembly and formation of LMS when the motif was assembled in buffer containing combinations of Ba^2+^ with other monovalent and divalent ions. A shift in the position of the LMS band is observed when divalent ions are present with Ba^2+^ in the annealing solution. (b) Shift in the relative mobilities of the LMS induced by the different concentrations of mono- or divalent ions when used in combination with Ba^2+^, compared to the mobility of the LMS in solutions containing only Ba^2+^. (c) Effect of different metal ions on the relative mobility of the LMS at different concentrations of Ba^2+^. The effect of divalent ions on retardation of LMS is more pronounced at lower concentrations of Ba^2+^ while monovalent ions have a negligible effect.

To assess whether the phenomenon of LMS formation is exclusive to DAO structures, we tested a simple duplex and another type of DNA double crossover motif with antiparallel strand crossovers separated by an even number of half-turns (21 bp, abbreviated DAE) (Figure 4a and Figure S16). We assembled the structures in 1× TAE-Mg^2+^ and validated assembly using nondenaturing PAGE (Figure 4b). We then assembled the structures in buffer containing different concentrations of Ba^2+^ and ran them on a nondenaturing polyacrylamide gel as before to examine the *R*_f_ of the assembly products (Figure 4c, full gel images in Figure S17). As the concentration of Ba^2+^ increased, we observed the formation of LMS in both the duplex as well as the DAE motif, indicating that at higher concentrations (above ∼20 mM), Ba^2+^ causes anomalous electrophoretic migration of other DNA structures as well. The gel mobility differences of the LMS in the duplex and DAE motif are similar to those observed in the DAO, although the amount of LMS is lower in these structures.

**Figure 4:**
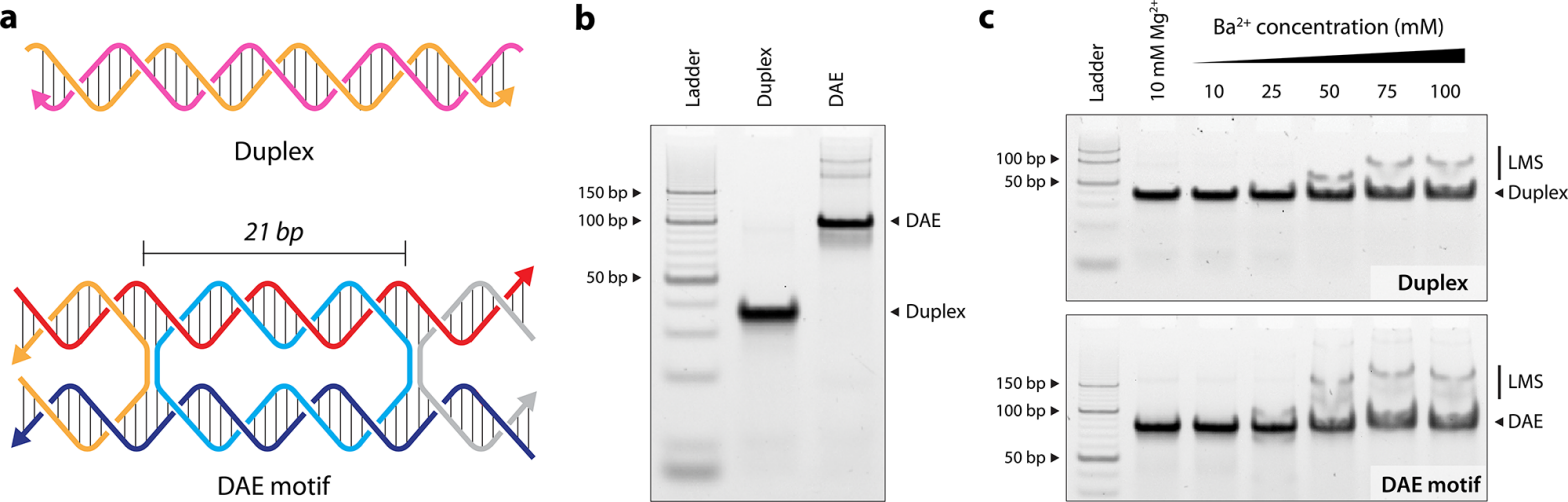
Formation of low mobility species in other DNA structures. (a) Design of the duplex and DAE motif. (b) Nondenaturing gel showing the assembly of the duplex and DAE motif. (c) Nondenaturing gel showing the Ba^2+^ induced formation of low mobility species in duplex DNA (top gel) and DAE motif (bottom gel).

While hydrogen-bonding interactions between the complementary regions of DNA strands are key to the self-assembly of DNA nanostructures, the complex electrostatic interactions between the ions in the solution and the DNA backbone also play an important role in the stability of these structures. As a highly charged polyanion, DNA requires cations to reduce its charge density in solution, and multivalent cations are known to influence the local structure of DNA as well as condense it.^15^ The ability of cations to only screen negative charges or induce condensation depends on the charge of the cation and the geometry of the groove.^16^ For example, thermal denaturation of 160 bp fragments of calf thymus DNA in the presence of divalent metal cations such as Ni^2+^, Mn^2+^, Ca^2+^, and Mg^2+^ led to aggregation of the DNA; however, Ba^2+^ and Sr^2+^ did not cause aggregation.^17^ The interactions between DNA and divalent alkali earth metal ions depend on steric and electronic factors. The ionic radii, the residence time of water molecules in the first solvation shell of the ion, and the coordination number influence the binding of these ions to DNA.^18^ In addition to interactions with the phosphate groups, the large divalent ions Sr^2+^ and Ba^2+^ are expected to interact with the nucleobases in the minor grooves (N3 of adenine, N3 of guanine, O2 of thymine, and O2 of cytosine) and major grooves (N7 and O6 atoms of guanine, N7 of adenine, and O4 of thymine).^19^ In double stranded DNA, Ba^2+^ ions may be involved in interstrand binding that bridges phosphate groups of the two complementary strands.^20^ Further, Ba^2+^ ions have been shown to bridge two side-by-side Z-DNA helices in a crystal by coordinating to the O6 and N7 atoms of two guanines simultaneously.^21^ These tendencies could be useful in creating reversible metal-mediated interactions between double stranded regions within DNA nanostructures. However, it is not clear whether such interactions are involved in the formation of LMS.

In the context of DNA nanostructures, divalent ions such as Mg^2+^ and Ca^2+^ are critical for folding of DNA strands and influence specific structural configurations of DNA branch points.^3,22^ In this study, we report the assembly of synthetic DNA motifs in Ba^2+^ and their anomalous electrophoretic migration in solutions containing >20 mM Ba^2+^. Electrophoretic mobility of DNA is dependent on the molecular weight, curvature of the duplex, ionic strength, charge density, and solution viscosity.^23−25^ Appearance of low mobility bands during DNA nanostructure assembly is usually associated with the formation of multimers or structures with unintended stoichiometric mixtures of DNA strands. In the present case, the incremental change in the position of the LMS band relative to the DAO motif band with increasing concentrations of Ba^2+^ indicates that the LMS is not a misfolded structure or a structure with incorrect strand stoichiometry. Further, CD spectra show that all of the LMS at different Ba^2+^ concentrations still retained a B-DNA conformation. Our results show that this phenomenon is unique to Ba^2+^ among the alkali earth metal ions. Assembly of the DAO motif in high concentrations (up to 100 mM) of other monovalent (Na^+^, K^+^, and Li^+^) and divalent ions (Mg^2+^, Ca^2+^, and Sr^2+^) did not show the formation of the LMS. However, divalent ions influenced the formation of LMS when combined with Ba^2+^, showing a complex synergistic activity of these ions with Ba^2+^. Our previous work showed that divalent transition metal ions such as Zn^2+^, Ni^2+^, and Cu^2+^ destabilize the DAO structure even at 10 mM concentrations,^6^ but it is still unknown if other less-commonly used counterions such as Mn^2+^ and Co^3+^ can generate complexes with retarded electrophoretic mobility. Metal ions such as Na^+^ and Mg^2+^ are routinely used to assemble various DNA nanostructures, while other ions such as K^+^ and Ca^2+^ have also been used. Although there are some reports of Ba^2+^ complexes with DNA, Ba^2+^ is rarely used to construct DNA nanostructures, probably because of its limited physiological relevance.^26−28^

Ba^2+^ induced the formation of electrophoretically retarded complexes not only with the DAO motif but also with simple duplexes and other crossover structures such as the DAE motif, suggesting that this phenomenon can potentially be applied to different types of DNA structures. It would be interesting to further analyze how the nanostructure influences LMS formation and to study this phenomenon in larger and more complex DNA assemblies containing multihelix bundles such as those constructed using the DNA origami method. Overall, our results are useful to understand the impact of Ba^2+^ on DNA nanostructure assembly and gain mechanistic insights into the formation of DNA nanostructures in different ionic compositions. Such information would be useful in applications involving the exposure of DNA nanostructures to high concentrations of multiple metal ions and to properly characterize DNA nanostructures employed under such atypical conditions.

## Supporting Information Available

Experimental methods, additional results, and DNA sequences used (PDF)
