# Supplementary material for: Barium Concentration-Dependent Anomalous Electrophoresis of Synthetic DNA Motifs
Source: ACS Appl Bio Mater. 2024 Apr 18;7(5):2704–9. doi: 10.1021/acsabm.4c00274 (PMC11110055; doi:10.1021/acsabm.4c00274)
Supplement: Supplementary file 1 — mt4c00274_si_001.pdf [file mt4c00274_si_001.pdf]

Barium concentration-dependent anomalous electrophoresis of synthetic DNA motifs

Bharath Raj Madhanagopal, Arlin Rodriguez, Mireylin Cordones and Arun Richard Chandrasekaran*

The RNA Institute, University at Albany, State University of New York, Albany, New York 12222, USA.

*Correspondence: arun@albany.edu

MATERIALS AND METHODS

Preparation of DNA complexes

DNA strands were purchased from Integrated DNA Technologies (IDT) with standard desalting and were used without further purification. To assemble the DNA complexes, component DNA strands (**Table S1**) were mixed in 1× tris-acetate EDTA (1× TAE) buffer containing 40 mM Tris base (pH 8.0), 20 mM acetic acid, 2 mM EDTA and the specified amounts of metal salt. DAO and DAE motifs were annealed in a thermal cycler with the following steps: 90 °C for 3 minutes, 65 °C for 20 minutes, 45 °C for 20 minutes, 37 °C for 30 minutes, 20 °C for 30 minutes and the solution was then cooled to 4°C. The samples were left to stand at 20 °C for 2 h before gel analysis. Duplex DNA was annealed from 90 °C to 20 °C over 30 minutes. Metal salts barium chloride ($BaCl_2$), lithium chloride (LiCl), magnesium chloride ($MgCl_2$), potassium chloride (KCl), sodium chloride (NaCl), and strontium chloride ($SrCl_2$) were purchased from Sigma-Aldrich with a purity >99%.

Non-denaturing polyacrylamide gel electrophoresis

Annealed DNA samples were mixed with 10× loading dye containing bromophenol blue and glycerol and loaded in polyacrylamide gels containing 19:1 acrylamide solution (National Diagnostics) and 1× TAE buffer. Gels were run at 4 °C with 1× TAE as the running buffer (without any metal ion) and stained with 0.5× GelRed (Biotium) in water for 20 min in dark and destained in water for 10 min. Gels were imaged on a Bio-Rad Gel Doc XR+ imager using the default settings for GelRed with UV illumination and analyzed using ImageLab software (Bio-Rad).

R_f analysis

Relative mobility (R_f) of the bands were measured using ImageLab software (BioRad) as the ratio of distance travelled by the band in the gel and the length of the gel. In Figure 1, The Y-axis represents the average of R_f measured in three replicate gels. In Figure 3b, the color intensity in the heat map represents the percentage shift in the Rf of LMS at different concentrations of Ba^{2+} and other metal ions with respect to the R_f of the LMS when only Ba^{2+} is present in the solution.

$$\%\ R_f \text{ shift in metal ion} = \%\ R_f \text{ of LMS at 0 mM } M^+ - \%\ R_f \text{ of LMS at each } M^+ \text{ concentration}$$

In Figure 3c, the Y-axis in the plots represent the % R_f of the LMS with respect to the mobility of the control DAO band (prepared with 10 mM Mg^{2+}).

$$\text{Percentage relative mobility} = \frac{R_f \text{ LMS at each concentration of } M^+}{R_f \text{ of DAO band in the control in that gel}} \times 100$$

Circular dichroism (CD) spectroscopy

CD spectra were collected on 1 μM DNA complexes assembled in 1× TAE buffer containing Mg^{2+} or Ba^{2+} at the concentrations specified in the text and Figure 2b. Experiments were performed on a Jasco-815 CD spectrometer at room temperature in a quartz cell with a 1 mm path length. CD spectra were collected from 360 to 200 nm with a scanning speed of 100 nm/min, bandwidth of 1 nm, and digital integration time was set at 1 s. The average of three accumulations were recorded.

Thermal melting studies

UV thermal melting studies were performed using 1 μM DNA complexes assembled in 1× TAE containing Mg^{2+} or Ba^{2+} at the concentrations specified in the text and Figure 2c. Experiments were performed in a Cary 3500 UV-Visible Spectrophotometer equipped with a temperature controller. Melting curves were acquired at 260 nm by heating and cooling from 20 °C to 90 °C at a rate of 0.5 °C/min. The data were fitted to the Boltzmann function using OriginPro. Melting temperatures were determined from the first derivative of the fitted melting curves.

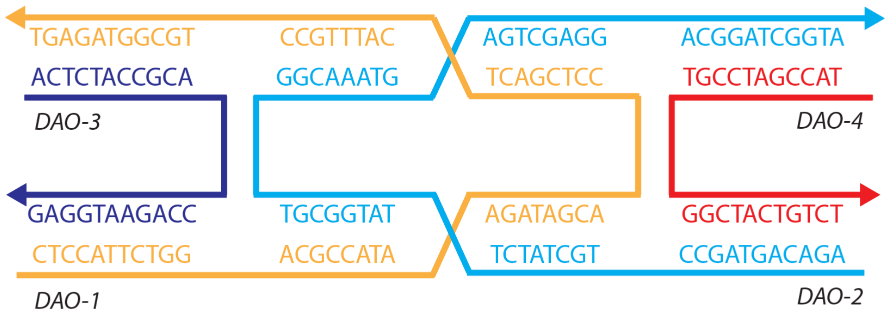

Figure S1. Sequence design of the DAO motif.

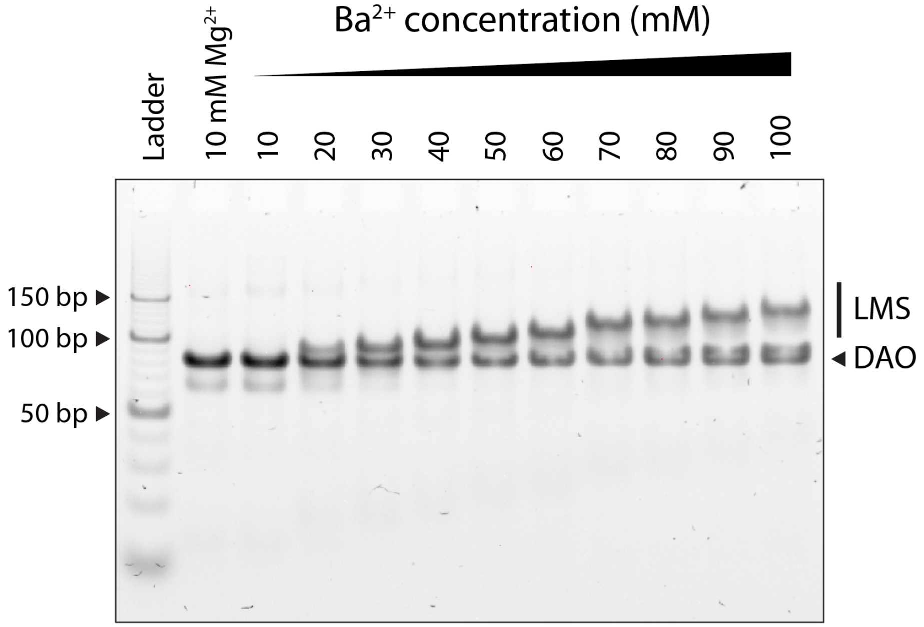

Figure S2. Nondenaturing gel showing the formation of LMS in 250 nM DAO assembled in 1× TAE containing different concentrations Ba^{2+}. Full image of gel shown in Figure 1d.

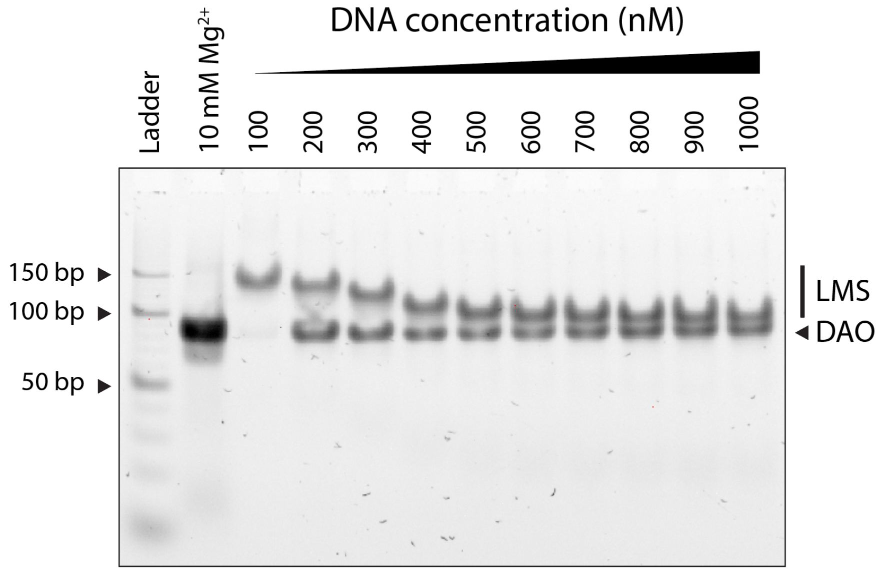

Figure S3. Nondenaturing gel and relative mobility analysis of DAO and LMS at fixed Ba^{2+} concentration (100 mM) and different DNA concentrations. Full image of gel shown in Figure 1e.

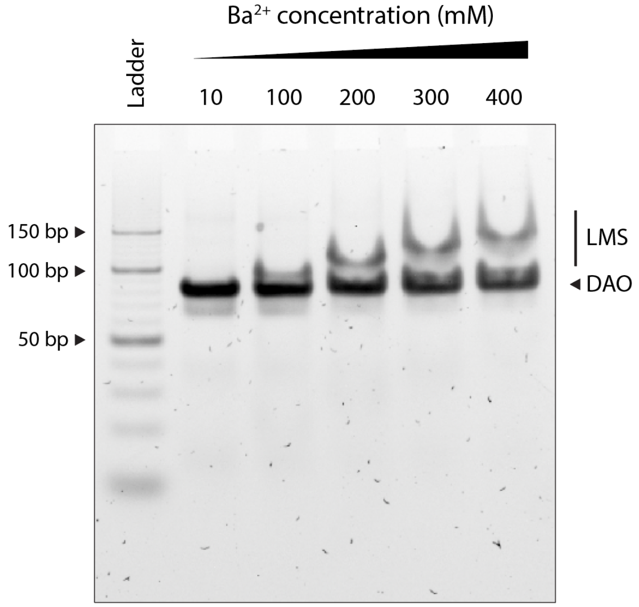

Figure S4. Gel showing the formation of the low mobility band at higher concentrations of Ba^{2+} when the DAO concentration is 1 µM.

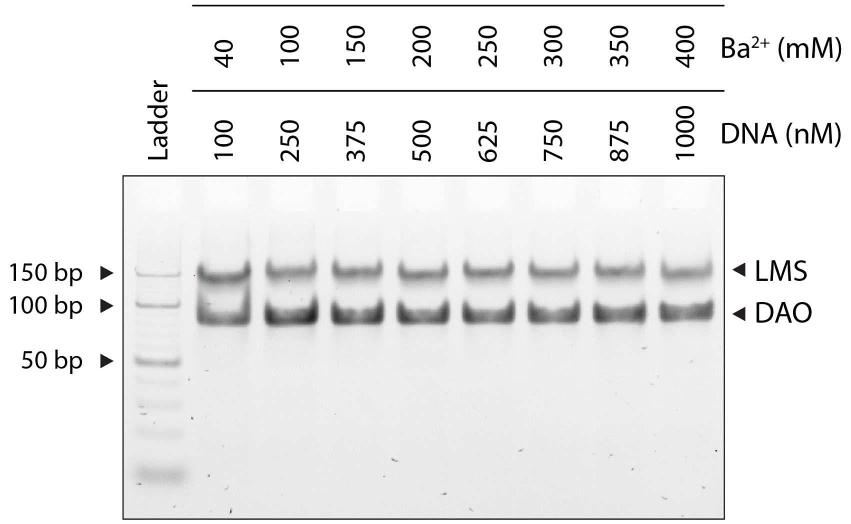

Figure S5. Nondenaturing gel and relative mobility analysis of DAO and LMS at fixed [DAO]:[Ba^{2+}] ratios. Full image of gel shown in Figure 1f.

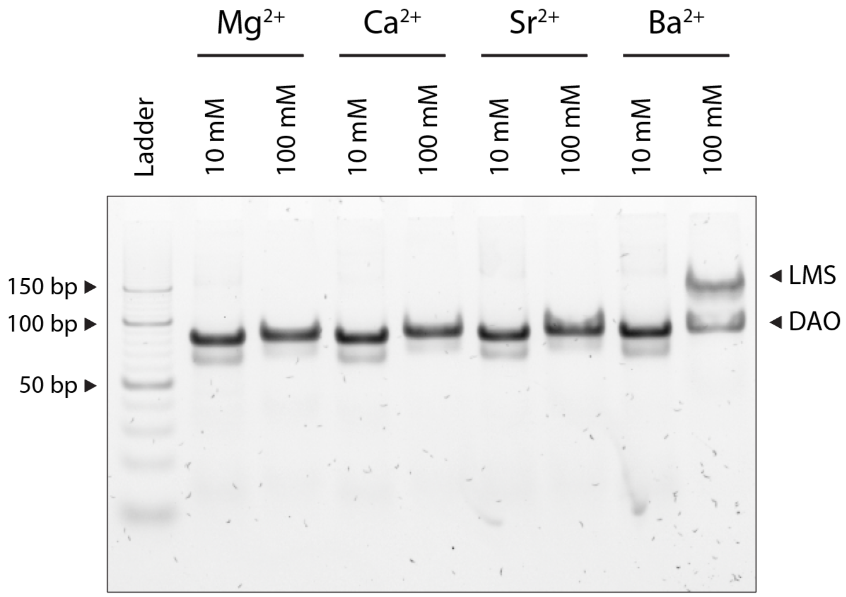

Figure S6. Gel showing the absence of the low mobility band at higher concentrations of Mg^{2+}, Ca^{2+} and Sr^{2+}.

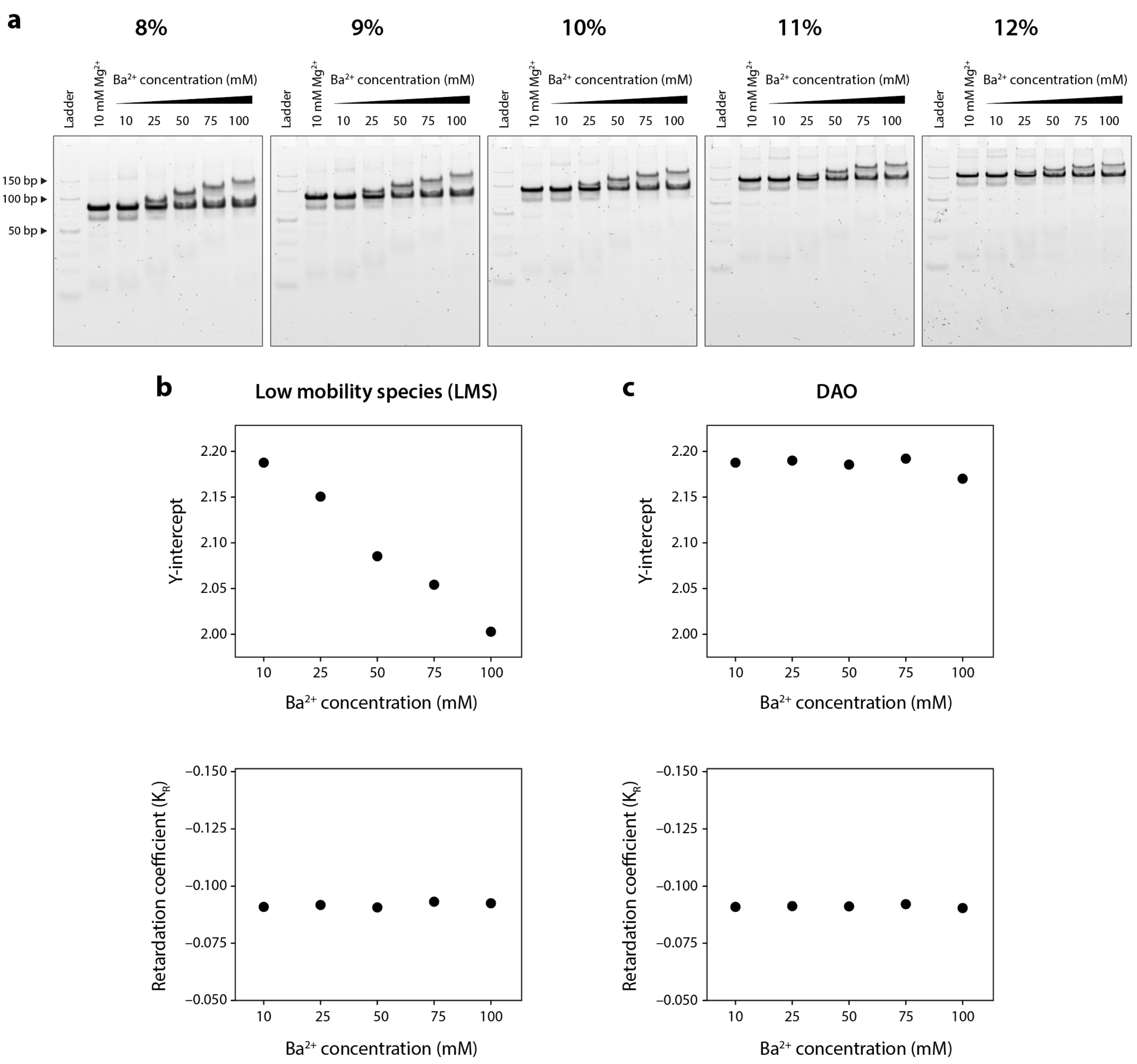

Figure S7. (a) Gel images used for Ferguson plot analysis. (b) Y-intercept and retardation coefficient (slope) for LMS. (c) Y-intercept and retardation coefficient (slope) for DAO.

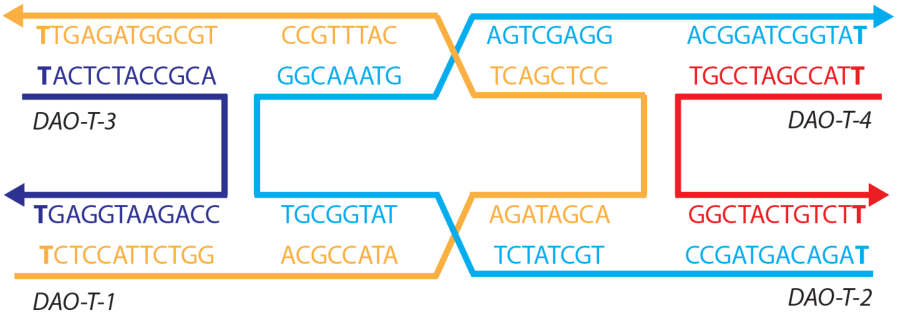

Figure S8. Sequence design of the DAO motif with terminal Ts.

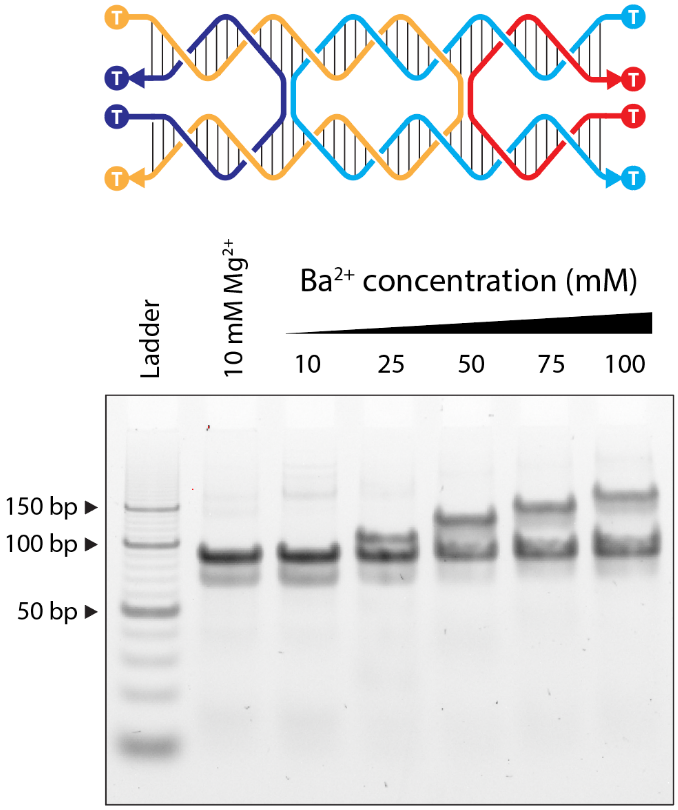

Figure S9. Nondenaturing gel showing the formation of LMS in DAO with terminal thymines assembled in 1× TAE containing 25-100 mM Ba^{2+}. Full image of gel shown in Figure 2d.

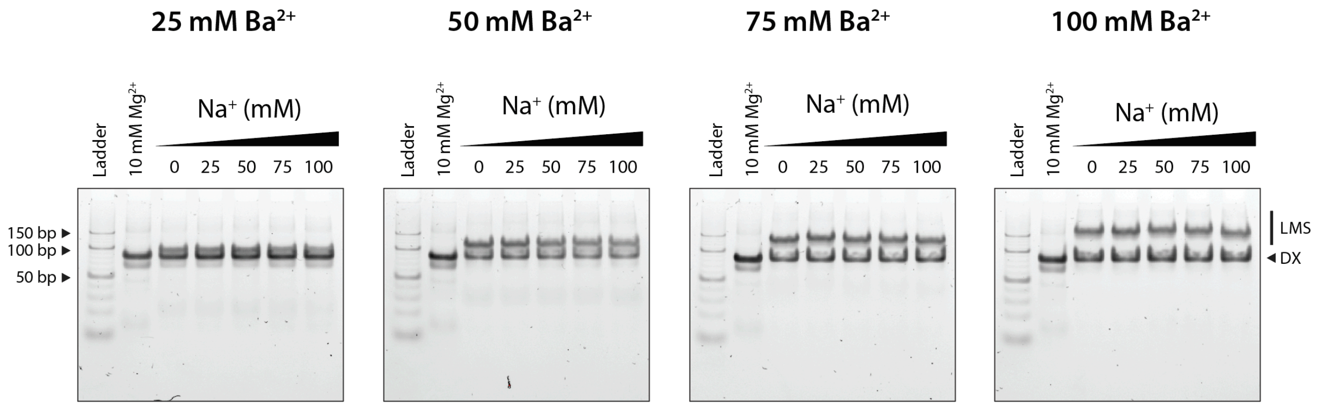

Figure S10. Non-denaturing gels showing DAO assembly and formation of LMS when the motif was assembled in buffer containing combinations of Ba^{2+} with different concentrations of Na^{+}. Full images of gels shown in Figure 3a.

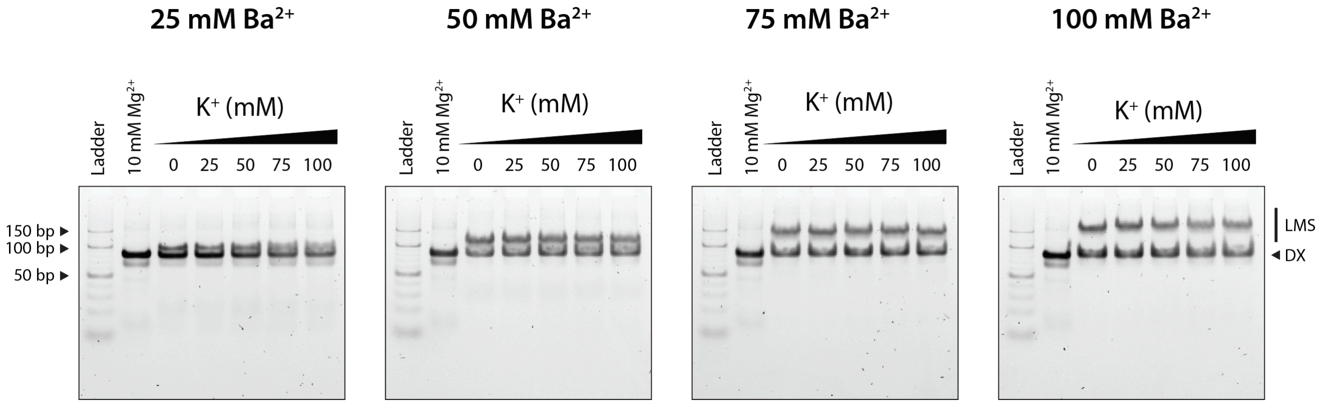

Figure S11. Non-denaturing gels showing DAO assembly and formation of LMS when the motif was assembled in buffer containing combinations of Ba^{2+} with different concentrations of K^{+}. Full images of gels shown in Figure 3a.

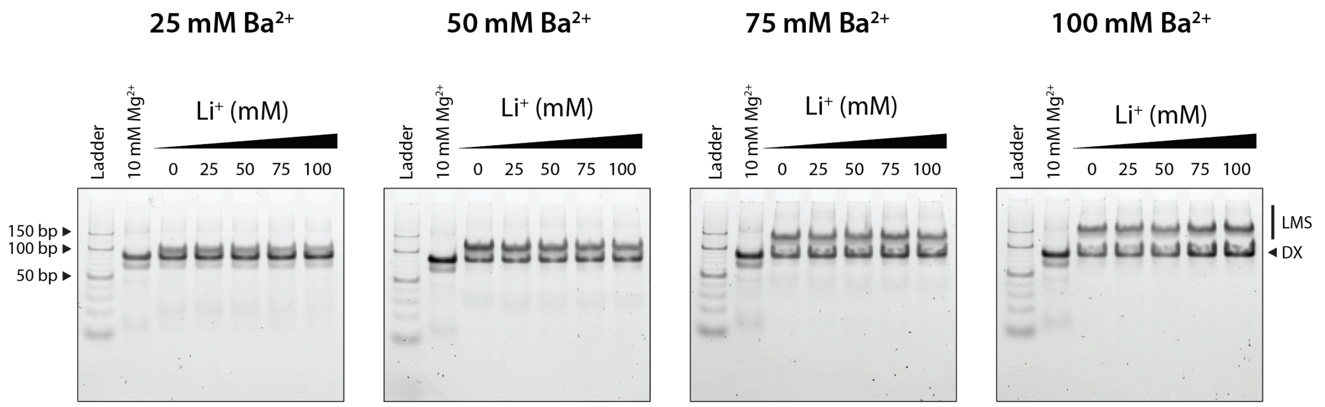

Figure S12. Non-denaturing gels showing DAO assembly and formation of LMS when the motif was assembled in buffer containing combinations of Ba^{2+} with different concentrations of Li^{+}. Full images of gels shown in Figure 3a.

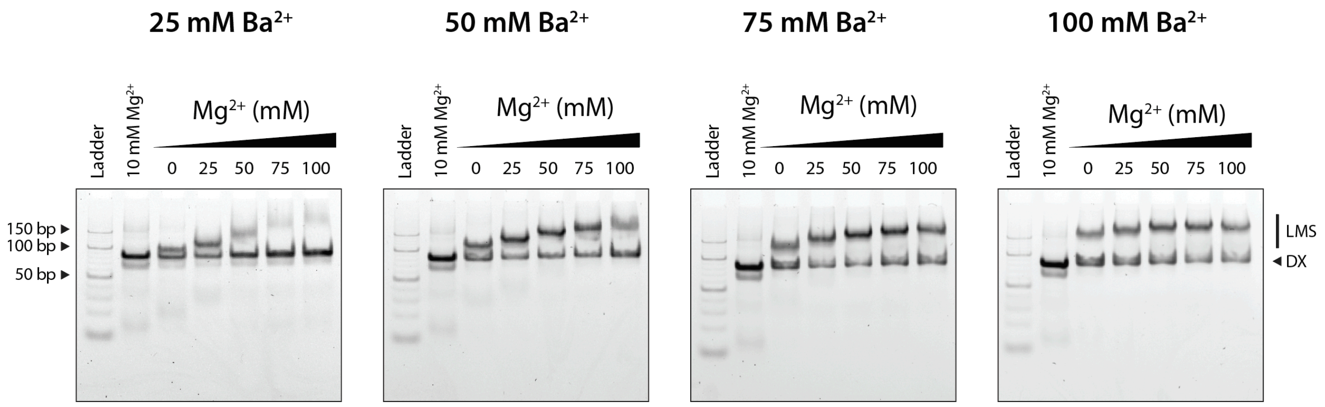

Figure S13. Non-denaturing gels showing DAO assembly and formation of LMS when the motif was assembled in buffer containing combinations of Ba^{2+} with different concentrations of Mg^{2+}. Full images of gels shown in Figure 3a.

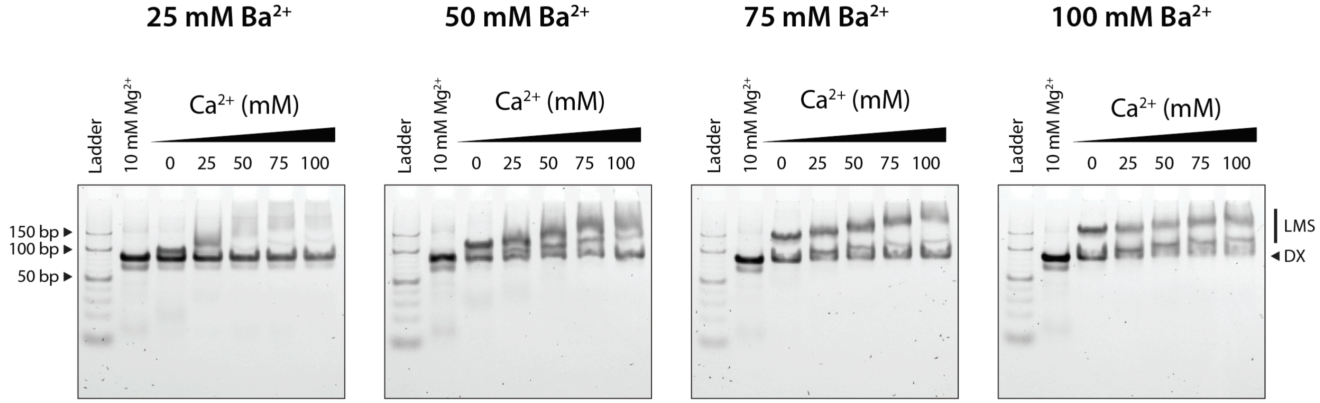

Figure S14. Non-denaturing gels showing DAO assembly and formation of LMS when the motif was assembled in buffer containing combinations of Ba^{2+} with different concentrations of Ca^{2+}. Full images of gels shown in Figure 3a.

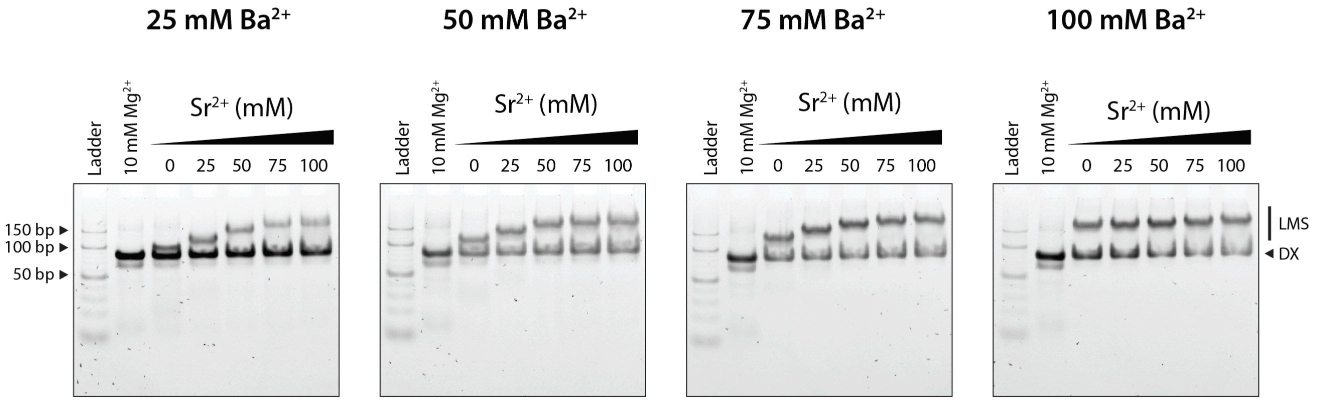

Figure S15. Non-denaturing gels showing DAO assembly and formation of LMS when the motif was assembled in buffer containing combinations of Ba^{2+} with different concentrations of Sr^{2+}. Full images of gels shown in Figure 3a.

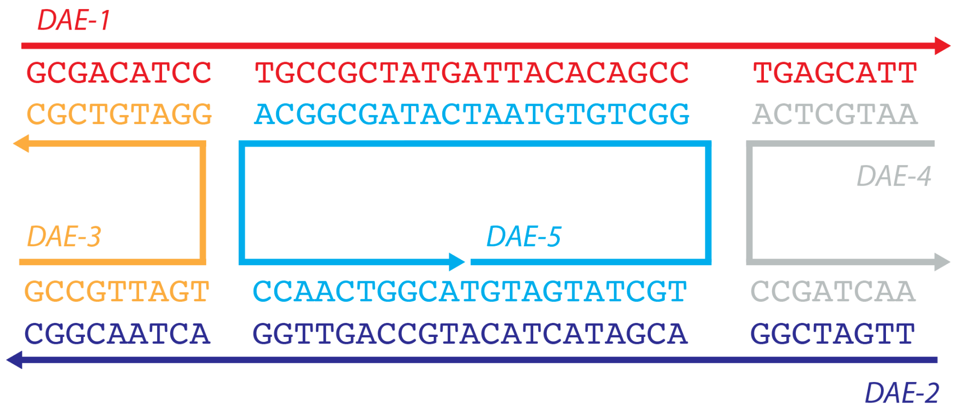

Figure S16. Sequence design of the DAE motif.

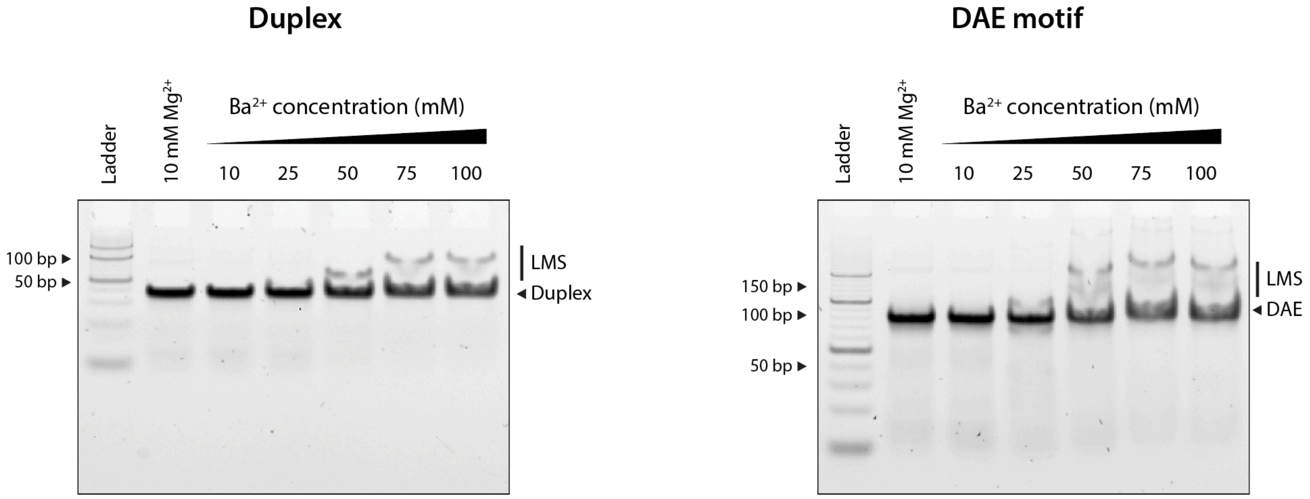

Figure S17. Non-denaturing gel showing the Ba^{2+} induced formation of low mobility species in duplex DNA (left gel) and DAE motif (right gel). Full images of gels shown in Figure 4c.

Table S1. DNA sequences used.

Strand name	Sequence (5′ to 3′)
DAO1	CTCCATTCTGGACGCCATAAGATAGCACCTCGACTCATTTGCCTGCGGTAGAGT
DAO2	AGACAGTAGCCTGCTATCTTATGGCGTGGCAAATGAGTCGAGGACGGATCGGTA
DAO3	ACTCTACCGCACCAGAATGGAG
DAO4	TACCGATCCGTGGCTACTGTCT
DAO-T-1	TCTCCATTCTGGACGCCATAAGATAGCACCTCGACTCATTTGCCTGCGGTAGAGTT
DAO-T-2	TAGACAGTAGCCTGCTATCTTATGGCGTGGCAAATGAGTCGAGGACGGATCGGTAT
DAO-T-3	TACTCTACCGCACCAGAATGGAGT
DAO-T-4	TTACCGATCCGTGGCTACTGTCTT
Duplex 1	ACTCTACCGCAGGCAAATGAGTCGAGGACGGATCGGTA
Duplex 2	TACCGATCCGTCCTCGACTCATTTGCCTGCGGTAGAGT
DAE1	GCGACATCCTGCCGCTATGATTACACAGCCTGAGCATT
DAE2	TTGATCGGACGATACTACATGCCAGTTGGACTAACGGC
DAE3	GCCGTTAGTGGATGTCGC
DAE4	AATGCTCACCGATCAA
DAE5	TGTAGTATCGTGGCTGTGTAATCATAGCGGCACCAACTGGCA

Table S2. Melting temperatures of DAO motif annealed in 1× TAE buffer containing Mg^{2+} or Ba^{2+} (DAO concentration 1 μM).

Ion concentration	Tm (°C)	ΔTm (°C)
10 mM Mg^{2+}	68.0	-
10 mM Ba^{2+}	63.1	4.9
100 mM Ba^{2+}	65.6	2.4
200 mM Ba^{2+}	65.2	2.8
300 mM Ba^{2+}	63.9	4.1
400 mM Ba^{2+}	62.7	5.3
